# Double vs single internal thoracic artery harvesting in diabetic patients: role in perioperative infection rate

**DOI:** 10.1186/1749-8090-3-35

**Published:** 2008-06-23

**Authors:** Marco Agrifoglio, Matteo Trezzi, Fabio Barili, Luca Dainese, Faisal H Cheema, Veli K Topkara, Chiara Ghislandi, Alessandro Parolari, Gianluca Polvani, Francesco Alamanni, Paolo Biglioli

**Affiliations:** 1 Department of Cardiovascular Surgery, Centro Cardiologico Monzino, University of Milan, Via Parea 4, 20138 Milan, Italy; 2 Division of Cardiothoracic Surgery, College of Physicians and Surgeon of Columbia University – New York Presbyterian Hospital, Columbia University Medical Center, Milstein Hospital Building, 7GN-435, 177 Fort Washington Avenue, New York, NY 10032, USA

## Abstract

**Background:**

The aim of this prospective study is to evaluate the role in the onset of surgical site infections of bilateral internal thoracic arteries harvesting in patients with decompensated preoperative glycemia.

**Methods:**

81 consecutive patients with uncontrolled diabetes mellitus underwent elective CABG harvesting single or double internal thoracic arteries. Single left ITA was harvested in 41 patients (Group 1, 50.6%), BITAs were harvested in 40 (Group 2, 49.4%). The major clinical end points analyzed in this study were infection rate, type of infection, duration of infection, infection relapse rate and total hospital length of stay.

**Results:**

Five patients developed sternal SSI in the perioperative period, 2 in group 1 and 3 in group 2 without significant difference. All sternal SSIs were superficial with no sternal dehiscence. The development of infection from the time of surgery took 18.5 ± 2.1 and 7.3 ± 3.0 days for Groups 1 and 2 respectively. The infections were treated with wound irrigation and debridement, and with VAC therapy as well as with antibiotics. The VAC system was removed after a mean of 12.8 ± 5.1 days, when sterilization was achieved. The overall survival estimate at 1 year was 98.7%. Only BMI was a significant predictor of SSI using multivariate stepwise logistic regression analysis (Odds Ratio: 1.34; 95%Conficdence Interval: 1.02–1.83; p value: 0.04). In the model, the use of BITA was not an independent predictor of SSI.

**Conclusion:**

CABG with bilateral pedicled ITAs grafting could be performed safely even in diabetics with poor preoperative glycaemic control.

## Background

The role of diabetes in the onset of surgical site infections (SSI) is well established, especially in patients with uncontrolled-glycaemia levels[1]. It represents a predisposing factor, as it leads to microvascular alterations and decreased wound healing [2,3]. Moreover, it interferes with the immunological response against infection. The diabetic pattern synergically increases the impact of other risk factors, such as obesity, gender, age, tobacco use, respiratory disease, or local impairment of vascularization [1,4].

The harvesting of internal thoracic artery (ITA) for coronary artery bypass grafting (CABG) leads to an acute decrease of the sternal circulation [5]. It was demonstrated to reduce significantly the perfusion of the hemi-sternum in the perioperative period, while after few weeks, collateral circulation can supply it [5-7]. Hence, the sternal wound is exposed to increased risk of dehiscence and SSI by its nature, especially when other risk factors are present.

Starting from this issue, the harvesting of bilateral internal thoracic arteries (BITAs) has been considered an adjunctive risk factor for postoperative sternal wound complication [8] and consequently it has been contraindicated in diabetic patients at high risk. If the use of BITAs in diabetics is associated with a higher rate of morbidity and mortality, one could question the wisdom of using bilateral ITA grafting for a potential long-term benefit that would be neutralized by an increased early risk [9-12]. The aim of this prospective non-randomized study was to evaluate the role of BITAs versus single ITA grafting in a population of diabetic patients with decompensated preoperative glycemia undergoing on-pump CABG with no associated procedures.

## Methods

From January 2006 and July 2006, 81 consecutive patients with uncontrolled diabetes mellitus underwent elective CABG harvesting single or double internal thoracic arteries (27.5% of all CABGs in the same period).

Single left ITA was harvested in 41 patients (Group 1, 50.6%), BITAs were harvested in 40 (Group 2, 49.4%). The choice of single or double ITAs was guided by patient's age, clinical status and surgeon's preference. In all cases, ITAs were dissected non-skeletonized from the thoracic wall, along with internal thoracic veins, muscles, and fascia.

The two groups were compared with regards to their baseline characteristics, operative factors, and clinical outcomes. Baseline characteristics included age, gender, primary cardiac pathology, presence of hypertension, diabetes, chronic obstructive pulmonary disease (COPD), obesity, Body Mass Index (BMI), renal failure, peripheral vascular disease, hypercholesterolemia, smoking history, previous cardiac operations, previous myocardial infarction, the "European System for Cardiac Operative Risk Evaluation" (EuroSCORE) [13-15]. The operative factors examined were type of surgery, duration of operation, re-exploration for bleeding, the amount of postoperative bleeding, the number of transfused patients, the number of transfusions, incidence of postoperative intubation, and intra-aortic balloon pump (IABP) insertion during or after surgery, intensive care unit (ICU) stay. No different surgical techniques were used in the 2 groups and only the number of drainage tubes differed. Patients in Group 2 received 2 adjunctive drainages, one placed in the anterior mediastinum and one between sternum and pectoralis fascia.

In perioperative period, the patients' blood glucose levels were monitored and insulin treatment was administered, if necessary, with the goal of keeping blood sugar levels at or below the safe limit of 200 mg/100 ml [16].

The major clinical end points analyzed in this study were infection rate, type of infection, duration of infection, infection relapse rate, rate of hospital readmission, duration of antibiotic use, and total hospital length of stay. The presence of sternal SSIs was determined using the Center of Disease Control (CDC) criteria [17]. Management of organ/space sternal SSIs in all patients started with an initial empirical antimicrobial therapy. The subsequent therapy was dynamically guided by the antibiotic susceptibility tests. Antibiotic therapy was managed by an infectious disease physician, together with the cardiac team. The treatment of organ/space sternal SSIs was previously described [18]. Superficial sternal SSIs was performed in the same fashion through reopening of the surgical suture, debridement of all infected and avascular tissue, curettage of the cutaneous/subcutaneous tissues and daily local debridement with antiseptic irrigation until the site sterility was reached. Vacuum-assisted wound closure (VAC, KCl Inc., San Antonio, TX) therapy was applied in all sternal SSI. VAC therapy is a non-invasive active therapy, based on the application of negative pressure by controlled suction to the wound surface [19,20]. It is known to enhance granulation and wound contraction. No hyperbaric oxygen therapy was used in this group. Antibiotics were discontinued when no clinical signs or symptoms of infection persisted and when 2 cultures obtained from the wound were found to be negative. Infection relapse was diagnosed by isolation of organisms from an aseptically obtained culture and clinical data according to the Center of Disease Control (CDC) criteria. The sternal wound was closed soon after sterilization.

Data collection was prospective and preoperative, perioperative, and postoperative data were obtained from our institutional database and reviewed using a standard data collection form. Patients were regularly followed up at 3, 6, and 12 months. This study had the approval of our institutional ethics committee, and written informed consent was obtained from every patient by the senior investigator in accordance with institutional guidelines.

### Statistical analysis

Categorical variables are represented as frequency distributions and single percentages. Values of continuous variables are expressed as a mean ± standard deviation (SD).

Continuous variables were compared using an independent t-test, and categorical variables were compared by χ^2 ^and Fisher's exact test, where appropriate.

Significant predictors of sternal SSI were investigated by a stepwise logistic regression analysis on preoperative, operative and postoperative factors.

Actuarial life table estimates were constructed using the Kaplan-Meier method.

For all analyses, two-sided p < 0.05 was considered significant. Statistical analysis was performed using SPSS 13.0 software (SPSS, Chicago, IL, USA).

## Results

Preoperative data are represented in Table 1. Group 2 had a significant lower age, better EF and EUROSCORE. It had been related to the indications for performing BITA harvesting, younger age and good preoperative clinical status being an indication to BITA CABGs. There were no significant differences in gender, number of diseased vessels, and comorbidities between groups. Medical treatment of diabetes was achieved preoperatively with insulin in 14 patients from Group I versus 14 patients from Group II and with oral hypoglycemic agents in 27 patients from Group I versus 26 patients from Group II (p = 0.896). Preoperative blood fasting glucose and glycated hemoglobin were similar in the two groups.

**Table 1:** Preoperative Clinical Characteristics

	**Group 1**	**Group 2**	***p*-value**
**Number of Patients**	41 (50.6%)	40 (49.4%)	
**Mean Age (Years)**	66.5 ± 8.0	62.3 ± 7.2	0.016
**Gender**			0.963
Male	35 (85.4%)	34 (85.0%)	
Female	6 (14.6%)	6 (15.0%)	
**Cardiac Pathology**			
Coronary Artery Disease	41 (100%)	40 (100%)	
Other	0 (0%)	0 (0%)	
**Number of Diseased Vessels**	2.8 ± 0.4	2.9 ± 0.3	0.089
Two-vessel disease	10 (24.4%)	4 (10.0%)	0.140
Three-vessel disease	31 (75.6%)	36 (90.0%)	
**Hypertension**	33 (80.5%)	25 (62.5%)	0.088
**Diabetes**	41 (100.0%)	40 (100.0%)	1.000
Non-Insulin-Dependent	34 (82.9%)	34 (85.0%)	0.799
Insulin-Dependent	7 (17.1%)	6 (15.0%)	
Preoperative fasting plasma glucose (mg/dL)	155.6 ± 53.7	148.5 ± 43.1	0.511
Glycated haemoglobin (%)	8.3 ± 0.9	8.6 ± 1.0	0.876
**Body Mass Index**	27.9 ± 4.1	26.9 ± 3.1	0.235
**Body Surface Area (m^2^)**	1.8 ± 0.1	1.8 ± 0.2	0.814
**Chronic Obstructive Pulmonary Disease**	6 (14.6%)	4 (10.0%)	0.737
**Renal Failure**	5 (12.1%)	3 (7.5%)	0.712
**Serum creatinine (mg/dL)**	1.1 ± 0.2	1.0 ± 0.2	0.471
**Peripheral vascular disease**	10 (24.4%)	5 (12.5%)	0.253
**Previous stroke**	1 (2.4%)	0 (0.0%)	1.000
**C reactive protein (mg/dL)**	4.1 ± 5.7	3.5 ± 6.8	0.653
**Hypercholesterolemia**	27 (65.9%)	27 (67.5%)	1.000
**Smoking History**	26 (63.4%)	26 (65.0%)	1.000
**Previous Cardiac Surgery**	0 (0.0%)	0 (0.0%)	
**Previous myocardial infarction**	22 (53.7%)	11 (27.5%)	0.024
**Ejection fraction**	53.7 ± 11.4	58.2 ± 8.0	0.041
**EuroScore**	4.2 ± 2.7	2.3 ± 1.6	0.000
**Length of Hospital Stay prior to Surgery (Days)**	4.0 ± 3.0	3.6 ± 3.3	0.753
**Inotropic Drugs before Surgery**	2 (4.9%)	2 (5.0%)	1.000

Operative data are shown in Table 2. All patients in this study underwent on pump Coronary Artery Bypass Grafting (CABG). No additional cardiac procedures were performed in each case. The number of distal anastomoses were comparable in both groups but distal anastomoses with venous and arterial grafts were significantly higher in group 1 and 2 respectively. Even the cross-clamping time, the CPB time and the duration of operation were significant higher in Group 2, reflecting the technical difficulties related to the RITA grafting. Re-exploration for bleeding, transfusions, ICU stay and postoperative intubation were similar for both groups. IABP insertion during or after surgery was needed in no cases. None of the patients in either group had infections related to other organs or surgical sites. Glycaemia was strictly monitored and controlled in all patients, in the preoperative, perioperative and postoperative period.

**Table 2:** Operative and Perioperative Details

	**Group 1**	**Group 2**	**p value**
**Type of surgery**			
CABG	41 (100.0%)	40 (100.0%)	
On Pump	41 (100.0%)	40 (100.0%)	
**Number of ITA grafts used**			
One	41 (100.0%)	0 (0.0%)	0.000
Two	0 (0.0%)	40 (100.0%)	
**Number of distal anastomosis**	3.3 ± 0.9	3.2 ± 6.7	0.810
With vein grafts	1.9 ± 0.8	1.0 ± 0.5	0.000
With arterial grafts	1.3 ± 0.5	2.3 ± 0.5	0.000
**Duration of operation (min)**	245.8 ± 40.0	272.8 ± 44.2	0.005
**CPB time (min)**	97.0 ± 21.6	110.8 ± 21.1	0.005
**Cross-clamp time (min)**	66.7 ± 18.6	87.0 ± 18.2	0.000
**Re-exploration for bleeding**	0 (0.0%)	2 (5.0%)	0.241
**Postoperative bleeding (ml)**	513.9 ± 239.6	836.5 ± 1025.1	0.059
**Transfused patients**	14 (34.1%)	11 (27.5%)	0.632
Red blood cell units	0.9 ± 1.4	0.8 ± 2.0	0.893
**Postoperative intubation (hours)**	7.8 ± 6.7	7.0 ± 5.9	0.676
**Intensive Care Unit stay (days)**	2.4 ± 4.1	1.8 ± 0.7	0.311

5 patients developed sternal SSI in the perioperative period, 2 in group 1 and 3 in group 2 without significant difference (Table 3). All sternal SSIs were superficial with no sternal dehiscence. The wound bacteriology was also found comparable for both groups in order of *Staphylococcus epidermidis *and *Staphylococcus aureus*. The development of infection from the time of surgery took 18.5 ± 2.1 and 7.3 ± 3.0 days for Groups 1 and 2 respectively. No statistical analysis was performed considering the small number of patients who developed SSI. As describe above, the infections were treated with wound irrigation and debridement, and with VAC therapy as well as with antibiotics. The VAC system was removed after a mean of 12.8 ± 5.1 days, when sterilization was achieved.

**Table 3:** Details of sternal SSIs and clinical outcomes.

	**Group 1**	**Group 2**	**p value**
**Organ/space sternal SSIs**	0 (0.0%)	0 (0.0%)	
**Superficial sternal SSIs**	2 (4.9%)	3 (7.5%)	0.675
*Staphylococcus Aureus*	1	2	
*Staphylococcus Epidermidis*	1	1	
**Time from surgery to recognized infection (days)**	18.5 ± 2.1	7.3 ± 3.0	
**Duration of infection until sterilization (days)**	14.0 ± 4.2	13.3 ± 3.7	
**Infection relapse rate**	0 (0.0%)	0 (0.0%)	
**Total hospital length of stay (days)**	15.5 ± 3.5	19.0 ± 7.0	

At one-year follow-up, only 1 non-cardiac death occurred in Group 1. The overall survival estimate at 1 year was 98.7%. No further sternal SSI, dehiscence, or infection relapses occurred at 1 year follow-up.

All preoperative, operative and postoperative data were included in the multivariate analysis. Only BMI was a significant predictor of SSI using multivariate stepwise logistic regression analysis (Odds Ratio: 1.34; 95%Conficdence Interval: 1.02–1.83; p value: 0.04). In the model, the use of BITA was not an independent predictor of SSI.

## Discussion

The use of bilateral ITAs has been associated with better long-tern outcomes than single LITA graft. It improves survival and reduces the need for repeat revascularization [9,10], even in diabetic patients. Moreover, RITA graft has been reported to have a long-term patency rate similar to LITA graft [11]. Nevertheless, the perception of an increased risk of SSI has limited the BITA harvesting in patients with diabetes [12]. This prospective non-randomized study was undertaken to understand if bilateral pedicled ITAs in diabetics increase early risks despite the potential later benefit.

The two groups were similar and comparable for all preoperative, operative and postoperative parameters but age, previous myocardial infarction, EF and EUROSCORE. These differences reflect the indications to perform BITA grafting in our Institution. The use of BITA grafts significantly prolongs the total and cross-clamping time and it should be avoided in patients with higher operative risks in which aortic cross-clamping time should be shortened. Older patients are at higher operative risk, as well as patients with preoperative heart failure [21,22]. By its definition, the higher EUROSCORE of Group 1 confirms these data [13-15]. Nonetheless, these parameters are not considered risk factors for SSI and the should not alter the homogeneity of the groups [23].

The main outcome of this study is the similar rate of SSI of the 2 groups. The use of BITAs is considered by many Authors a significant risk factor for sternal dehiscence and mediastinal wound infection, as it leads to severe acute impairment of sternal blood supply. Single ITA harvesting leads to a 90% loss of hemisternum blood supply and bilateral ITAs mobilization can devascularize the entire sternum and compromise the healing of the sternal wound [5-7], especially in diabetic patients that are at higher risk of SSI. Diabetes is a well known risk factor for postoperative sternal SSI, but this study did not find significant differences between patients with single or bilateral ITAs harvesting. Even the multivariate analysis confirms that only BMI is an independent risk factor for SSI.

Pedicled bilateral ITA harvesting is traditionally considered hazardous in diabetics and many prefer skeletonized ITA mobilization, because skeletonized ITAs are associated with a higher residual sternal blood supply than that dissected as a pedicle [24]. Recent articles report that skeletonization of both ITAs decreases the risk of sternal infection in diabetic patients [25] and shows good short as well as long term cardiac outcome [26]. ITA skeletonization is technically more demanding and more time consuming than pedicled ITA harvesting; it has a surgical learning curve and there is no current data on long term patency rates [27]. Our data show that bilateral pedicled ITAs can be used in diabetic patients without increased morbidity and mortality. These results were achieved through various factors, including meticulous pedicled ITA harvesting, placement of 2 adjunctive drain tubes to keep the sternal wound dry in the postoperative period, careful wound closure, especially in obese patients and continuous and aggressive blood glucose monitoring in the postoperative period.

Our postoperative infections were only superficial and complete healing and primary wound closure were always achieved; no sternal dehiscence and no deep sternum infection were observed in our cases.

## Limitations of this study

This study is prospective in nature but patients were not randomized. Outcomes have to be confirmed by randomized controlled trials in order to unmask eventual confounding factors. Moreover the dimension of the 2 groups is limited, as shown by the wide confidence interval.

## Conclusion

CABG with bilateral pedicled ITAs grafting could be performed safely even in diabetics with poor preoperative glycaemic control. However, in order to limit SSIs, these patients need a strict post-operative blood glucose control.

## Competing interests

The authors declare that they have no competing interests.

## Authors' contributions

MA conceived of the study, performed surgeries and participated in its design and helped to draft the manuscript.

MT participated in the study's design and coordination, collected data and drafted the manuscript

FB participated in the study's design and coordination, create the database, performed the statistical analysis and drafted the manuscript.

LD performed surgeries, collected data, participated in the study's design and coordination and drafted the manuscript.

FHC, VKT, CG participated in the study's design, helped to perform the statistical analysis and edited the manuscript.

AP, GP and FA participated in the study's design, helped to draft the manuscript and edited it.

PB coordinated the study and participated in its design.

All authors read and approved the final manuscript.
